# Using Natural Zeolite as a Feed Additive in Broilers’ Diets for Enhancing Growth Performance, Carcass Characteristics, and Meat Quality Traits

**DOI:** 10.3390/life13071548

**Published:** 2023-07-12

**Authors:** Mutassim M. Abdelrahman, Hani H. Al-Baadani, Mohammed M. Qaid, Maged A. Al-Garadi, Gamaleldin M. Suliman, Mohsen M. Alobre, Saud I. Al-Mufarrej

**Affiliations:** Animal Production Department, College of Food and Agriculture Sciences, King Saud University, Riyadh 11451, Saudi Arabia; hsaeed@ksu.edu.sa (H.H.A.-B.); malgaradi@ksu.edu.sa (M.A.A.-G.); gsuliman@ksu.edu.sa (G.M.S.);

**Keywords:** broiler performance, carcass traits, clinoptilolite, meat and litter quality, physical size

## Abstract

Background: Using natural zeolites as a food additive in poultry diets offers an intriguing perspective. The objective of this study was to investigate the effects of zeolite addition and particle size on broiler performance, carcass characteristics, meat quality, moisture of excreta and litter, and intestinal measurements during 35 days. Methods: A total of 560 1-day-old female Ross-308 broilers were divided into five treatment levels (0, 5, 10, 15, and 20 g zeolite/kg diet) (*n* = 16 replicates/treatment, *n* = 8 replicates /particle size of each treatment). Performance was calculated weekly. Carcass characteristics, meat quality, small intestine (SI) measurements, litter pH, and moisture content were determined on day 35. Results: Litter pH, breast redness, cooking loss, chewiness, total weight, and SI length were all affected by zeolite treatments (*p* < 0.05). Particle size had an impact on the gastric pH and texture analysis. Their interaction had an effect on color redness, litter pH, and cooking loss. Performance was unaffected by either the main or interaction effects. Conclusion: Zeolite as a feed additive may be useful in broiler diets, particularly large particles. The performance and production efficiency factor improved numerically (*p* > 0.05) with increasing zeolite doses up to 10 g zeolite/kg diet.

## 1. Introduction

Poultry litter is a combination of poultry excreta (manure), feathers, dander, bedding material (rice husks, shavings, etc.), spilled feed, water, and other solid and aqueous waste materials [1]. Moisture and quality of excreta/litter are related to poultry performance, health, and welfare, and may be the cause of environmental problems in the commercial poultry sector [2]. The need for safer natural alternatives for use in poultry farms requires the development of management practices to reduce or eliminate moisture contamination from excreta or litter in poultry farms [3,4,5]. Recently, there has been renewed interest in the use of clinoptilolite in biological applications to reduce ammonia emissions and odor from chicken houses by binding nitrogenous cations such as NH4+ and NH3 as a cost-effective feed additive and as a bedding material supplement in the poultry industry [4,6,7]. Natural zeolites (clinoptilolite) with a three-dimensional structure consisting of a microporous arrangement of silica and alumina tetrahedral called aluminosilicates. Due to their large surface area, they exhibit specific physicochemical properties such as ion exchange, adsorption, size exclusion framework properties, and catalytic properties [8,9]. The main role of zeolite is to trap molecules—such as when added to poultry and animal feeds—due to its crystalline structure, as well as its ability to gain or lose water and selectively exchange a wide range of cations without significant structural changes [3,10].

Zeolite contains a cationic material that protects tissues from the accumulation of toxic substances and affects the utilization of Ca and P [11]. The beneficial effects of zeolite include a higher content of Na, K, Al, Si, or Zn, which play a role in mineral metabolism and electrolyte balance and lead to better bone composition [12]. According to Miles and Henry [13], increased aluminosilicate content in the diet resulted in a linear decrease in fecal moisture. The addition of clinoptilolite to broiler bedding or feed reduced the moisture content of the bedding, which was attributed to the higher water absorption capacity of the material [14].

For example, clinoptilolite has been used to promote growth in a variety of animal species, including poultry, as well as in agriculture, veterinary medicine, hygiene, environmental protection, and animal nutrition [15,16,17]. The use of zeolite does not alter the performance of broiler chickens [18,19] or reduce bacterial contamination of the intestine [20] and is considered to be an immune modulating factor of the immune system [21]. The mechanism of this effect is not precisely known, but it could be due to a longer residence time of the feed in the stomachs of the chickens. This exposes nutrients to enzymatic action for a longer period, which may explain the effect of zeolite on the improved digestibility of some nutrients in chickens [17], pigs [15], and rats [16]. Zeolites such as hydrated aluminosilicates are used as detoxifying agents in humans and animals due to their specific binding of heavy metals, oxygen, and reactive oxygen species (ROS). Moreover, detoxification or free radical scavenging is believed to be responsible for their ability to reduce lipid peroxidation [15,22]. Few studies exist in the literature investigating the effects of the dosage and/or particles of the natural zeolite additive incorporated into broiler feed on broiler productivity. Moreover, the dosages used varied considerably, ranging from 0 to 30 g zeolite/kg diet [23,24,25], and the particle sizes of the zeolite used also varied considerably, ranging from 0.1 to 2.5 mm [23,26,27,28]; the results were inconsistent, inconclusive, and warrant further investigation. The novelty of this study is the proposal of a new approach by analyzing the dose and effect of particle size effect of zeolite incorporated into broiler feed to improve broiler growth performance, carcass traits, and meat quality. This work highlights a new perspective on the effects of different doses (0–20 g zeolite/kg diet) in two particle sizes (1–2 mm) on broiler growth performance, carcass traits, and meat quality. Each dietary supplement is expected to improve bird performance and/or health with the lowest dose, smallest particle size, and no side effects. Therefore, we chose different dosages and particles of natural zeolite to determine the best dosages and particles and to compare our results with those of previous studies. The null hypothesis (H_0_ = 0, if the *p* value > 0.05), states that natural zeolite has no effect on growth performance, carcass characteristics, or meat quality. The alternative hypothesis (H_A_ > 0, if the *p* value < 0.05), hypothesized that the inclusion of natural zeolite as a feed additive in the diet of broilers has a positive effect on broiler chickens by improving selected parameters. The question that arises is whether such an approach is useful in terms of broiler performance production and whether aspects such as carcass yield, meat quality, and excreta/litter quality can be adequately considered. Therefore, the objective of this study was to investigate the effects of a natural zeolite and particle sizes on broiler growth performance, carcass yield, meat quality, intestinal morphometrics, and fecal/litter quality.

## 2. Materials and Methods

### 2.1. Birds, Feeding, and Experimental Design

All procedures used in this study were approved by the Animal Ethics Committee of King Saud University, Riyadh, Saudi Arabia. In this experiment, 560 1-day-old female broiler chicks of commercial strain Ross-308 were used. Broiler chicks were purchased from a local hatchery and housed in electrically heated cages. Chicks were weighed, and randomly assigned to 1 of 10 treatments.

The experiment was divided into 5 zeolite treatment levels (0, 5, 10, 15, and 20 g zeolite/kg feed), *n* = 16 replicates per treatment, and 8 replicates of each particle size in each treatment in a completely randomized design. The doses chosen were based on Schneider, Almeida [23], who used 5 g of zeolite per kg of feed for broilers. Safaeikatouli et al. [24] added zeolite to broiler chickens’ feed at 15 and 30 g/kg of feed. Additionally, El-Garhy [25] added zeolite to the feed for laying hens at a concentration of 0, 10, 15, and 20 g/kg feed. On the other hand, the particle sizes of natural zeolite used in broiler feed ranged from 0.1 mm to 1 mm [23,26]. In addition, Uriyanghai [27] used zeolite with a size of 1–2.5 mm. Mery, Guerrero [28] also used zeolite particle sizes of 0.5, 1.0, and 2.0 mm.

The birds’ diets were formulated in accordance with the commercial practice of Saudi Arabia. The birds were fed starter (0–21 days) and finisher (22–35 days) diets (Table 1) based on corn and soybean meal (SBM); diets were formulated in a mashed form. However, consistent with previous studies, in this study the feeding program was a starter at 0–14 days [29,30,31] and a finisher at 15–35 days [29,31]. In another study, a starter diet was fed from day 1 to day 21, and a finisher diet was fed from day 22 to day 35 of the experiment [32]. Shuaib, Ullah [31] offered a starter diet (0–21 days) and a finisher diet (22–42 days), and so on. Natural zeolite powder was mixed with treatment feed according to the aforementioned basis. The average temperature in Riyadh, Saudi Arabia, during the mixing process was 26.4 °C, in a semi-warm, sub-humid climate [33]. Feed and water were provided ad libitum. All chickens were vaccinated against NDV, IBV, and IBDV. Birds received their initial and booster vaccinations according to the manufacturer’s recommendations and instructions (Fort Dodge Animal Health, Fort Dodge, IA, USA).

### 2.2. Performance Measurements

At hatching, birds from each treatment were weighed individually. For five weeks, chick weight and total feed intake (TFI) [34] were recorded weekly for each replicate of treatments. The average body weight gain (BWG), feed conversion ratio (FCR), and production efficiency factor (PEF) for each treatment were calculated weekly. The mortality rate of each replicate was recorded daily. 

### 2.3. Lymphoid Organs, Gizzard, Small Intestine, and Carcass Parameters

At the end of the experiment (day 35), five chickens were randomly selected and slaughtered to determine carcass and meat quality characteristics and total body weights. The slaughtered birds selected for sampling were slaughtered using the Islamic method of cutting the jugular vein, and the chicks were left for about two minutes after cutting the jugular vein to bleed out completely. Then, the carcasses of the birds were carefully opened, and the lymphoid organs (bursa, spleen, and thymus) were removed and weighed separately. In addition, the heart, pancreas, liver, abdominal fat, breast, leg, and whole weight of the carcasses, were collected, and their relative weights were recorded.

Along with the weights of the bird, the empty gizzards without proventriculus were also weighed and recorded. The pH of the gizzard contents of two birds was measured using a calibrated pH meter [35].

The total weight (g) and length (cm) of the small intestine were also determined. The length and weight of the SI parts (duodenum, jejunum, and ileum) were recorded of the total length and weight of the SI.

### 2.4. Meat Quality Parameters

The chicken breasts were sliced and weighed. The pH in the incisions on the cranial left side of the breast muscle was determined with a microprocessor pH meter. The mean pH of the breast of each carcass was calculated using two measurements. The CIELAB, or CIE L*, a*, b*, color system (1976), which indicates lightness, redness, and yellowness, was measured in two different areas of the inner surface of the muscle at the cranial position using a Chroma meter (Konica Minolta CR-400, Tokyo, Japan). After evaluating the pH and color quality, the breast samples were immediately frozen and stored at 20 °C for evaluation of cooking loss (CL) and shear force (SF). The same samples were thawed at 4 °C for 24 h before heating. The sample was placed in an electric commercial stainless steel grill oven (GR 28215; Kalorik, Miami Gardens, FL, USA), and cooked for approximately 20–30 min at 200 °C to an internal temperature of 70 °C. The internal temperature was determined by piercing a thermocouple thermometer probe into the center of each steak. A semi-analytical scale was used before and after cooking to measure the CL percentage. The CL was then calculated using the following equation as follows: CL = [(initial weight of cooked meat − final initial weight of cooked meat) × (100)] ÷ Initial weight of cooked meat. The cooked meat samples used to calculate CL were also used to determine tenderness or SF. The samples were then cooled to 22 °C before being cut into five pieces (2, 1, and 1 cm diameter), with the longest length, parallel to the length of the muscle fibers. A texture analyzer (TA-HD Stable Micro Systems Ltd., Godalming, UK) equipped with a Warner–Bratzler shear blade attachment was used to measure the maximum force (kg) when piercing the muscle. The maximum force (kg) perpendicular to the fibers was defined as SF. The crosshead was set at a speed of 120 mm/min.

Texture profile analysis (TPA) of the breast meat samples was also determined. The subsamples were obtained by scoring the samples parallel to the longitudinal direction of the muscle fibers using a handheld sampling device [36]. For TPA analysis, the same analyzer was used for SF analysis. However, equipped with a compression-plate attachment that compressed each sample twice by 80%. Hardness, cohesiveness, springiness, and chewiness were measured.

To determine the water-holding capacity (WHC), one sample (2.0 ± 0.2 g) was taken from the cranial part of the breast samples and weighed [37]. The duplicate samples were placed on filter paper on two acrylic plates and weighed with a 10 kg weight for 5 min. After reweighing the samples, the WHC value was calculated using the following equation: WHC (%) = 100 − [(Initial weight − Final weight /Initial weight) × 100].

A 4 g breast sample was crushed with scissors and homogenized with 40 mL of cold isolating myofibril fragmentation index (MFI) buffer using Ultra Turrax to determine MFI, as described by Suliman, Alowaimer [36]. A HACH DR/3000 S spectrophotometer was used to measure the absorbance of a 0.5 mg/mL solution, which was measured at 540 nm, and the MFI was calculated by multiplying the absorbance value by the dilution factor.

### 2.5. Quality (Structure) and Moisture Content of Litter (Bedding Material)

On day 35, the quality of the bedding for each replicate was evaluated by visual inspection, as described in Kheravii, Swick [38]. A composite sample of approximately 0.3 kg of litter was collected for each replicate. Each sample was accurately weighed before being dried in an oven at 105 °C for 24 h. Moisture content (MC) was determined according to the method described by Kheravii, Swick [38].

### 2.6. Statistical Analysis

The general linear model procedure of SAS 904 software [39] was used to analyze the data variables (by ANOVA table). In a completely randomized experimental design, experimental treatments were divided into 5 levels of zeolite treatments, with *n* = 16 replicates/treatment and 8 replicates of each particle size (1 mm and 2 mm) in each treatment. The data were analyzed for the main effects of natural zeolite levels, physical size, and their interactions. The model shown below was used:

$$\gamma_{ijk}=\mu +C_{i}+P_{j}+CP_{ij}+e_{ijk}$$

where *γ_ijk_* denotes a single observation; μ is the experimental mean; *C_i_* denotes the effect of *i*th clinoptilolite level; *P_j_* denotes the effect of the *j*th particle size; *CP_ij_* denotes the effect of *ij*th clinoptilolite level by physical size interaction; and *e_ijk_* denotes the random error. Duncan’s Multiple Range Test (DMRT) was used for comparisons of different means when significant (*p* ≤ 0.05). In addition, percent data were transformed with arcsine before analysis, and actual percent data are reported.

## 3. Results

### 3.1. Performance and Production Efficiency

Growth performance indicators are shown in Table 2 and Table 3. Natural zeolites, their physical size, and their interaction did not affect growth performance variables (BWG, TFI, and FCR) and PEF (*p* > 0.05) during the starter (d 1 to 21), finisher (d 22 to 35), or entire experimental period (d 1 to 35). However, increasing the zeolite dose to up to 10 g of zeolite per kg of feed resulted in a numerical linear trend of improvement in performance and PEF. In addition, increasing the zeolite particle size from 1 mm to 2 mm resulted in a numerical increase in performance and PEF (*p* > 0.05).

### 3.2. Carcass Measurements

Table 4 shows the carcass traits of 35-day-old broiler chickens. Natural zeolite treatments, particle size, or their interaction did not affect the relative weights of the heart, liver, gizzard, breast, legs, pancreas, or abdominal fat (*p* > 0.05). However, their interaction had an effect on the relative weight of the pancreas (*p* = 0.012). The percentage of dressing (*p* = 0.019) was influenced by the interaction between treatment and particle size, but not by treatments or particle size alone (*p* > 0.05).

### 3.3. Percentage Weights of Lymphoid Organs

As shown in Table 5, the treatments, particle size, or their interaction had no effect on the weight percentages of the bursa of the Fabricius, spleen, and thymus of broilers at 35 days of age (*p* > 0.05). However, the main effect of the treatments was on the proportional weight of the spleen, which decreased when zeolite was added compared with the control.

### 3.4. Physiochemical Properties of Breast Samples

The initial and final pH and temperature of breast samples from 35-day-old broiler chickens are shown in Table 6. The treatments, particle size, or interaction between them did not affect the initial or final pH (*p* > 0.05). However, the ultimate pH was significantly different due to the interaction between treatment and particle size (*p* < 0.0001).

The initial temperature was significantly affected by the treatments (*p* < 0.0001), particle size (*p* < 0.0001), and their interaction (*p* < 0.0001). The ultimate temperature was significantly different due to the treatments (*p* < 0.0001) but did not differ due to particle size and the interaction between treatment and particle size (*p* > 0.05). Initial and ultimate colors were presented in Table 7. The treatments, particle size, or even the interaction between them had no effect on the initial lightness and yellowness (*p* > 0.05). The treatments, as well as the interaction between treatment and particle size (*p* < 0.01), had an effect on the initial and ultimate redness. Initial and ultimate redness were decreased with increasing zeolite dose. The Initial and ultimate redness was not affected by particle size (*p* > 0.05). Ultimate yellowness was affected only by particle size (*p* < 0.01); the birds that received large particles showed more yellowness and lower lightness values of breast muscle.

### 3.5. Meat Quality Traits

Table 8 shows the meat quality of 35-day-old broiler breast meat. The treatments with natural zeolites, particle size, and their interaction had no effect (*p* > 0.05) on WHC%, MFI, and SF. However, the percentage of CL was influenced by the treatments with natural zeolites (*p* < 0.0001) and treatment–particle size interaction (*p* < 0.0001). Treatment 3 resulted in the greatest CL losses, while treatment 1 resulted in the least.

Table 8 also includes a texture profile analysis. The treatments had no effect on hardness, cohesiveness, chewiness, or springiness, nor did the interaction between treatment and particle size (*p* > 0.05). Treatments, on the other hand, had a significant effect on chewiness, with treatment 1 having the lowest value when compared to the control and the other treatments being the same.

Particle size had an effect on hardness (*p* = 0.007), springiness (*p* = 0.0004), and cohesiveness (*p* < 0.0001). Broiler chicken breast meat had less hardness, more springiness, and more cohesiveness in the large particle size group (2 mm).

### 3.6. Small Intestine Measurements

Table 9 shows the morphometric measurements of intestinal parts relative to the total weights and lengths of the SI in broilers at 35 days of age. Treatments and the interaction between the treatment and particle size affected the total weight and length of SI, but physical size had no effect (*p* > 0.05). Birds fed a 10 g zeolite/kg diet had higher total SI weight compared to the control group. Among the treatments, the birds fed 15 g of zeolite per kg of diet had the shortest SI length.

There were no effects of treatments, particle size, or their interaction on the percent weights and lengths of duodenum, jejunum, and ilium (*p* > 0.05). However, treatment had a significant effect on jejunum weight (*p* = 0.011). Birds fed a 5 g zeolite/kg diet had higher jejunal weight compared with the control and the other treatments. It was also found that the interaction of treatment and particle size had a significant effect on jejunal weight (*p* = 0.0004).

### 3.7. Litter Quality and Moisture Content

Table 10 shows the pH of GIT, the pH of the litter, and the moisture percentage of the litter contents of 35-day-old broiler chickens. There were no statistically significant differences in GIT pH due to treatment or treatment and particle size interaction (*p* > 0.05). However, significant differences were observed in the pH of GIT due to particle size (*p* = 0.043). The pH of GIT decreased with increasing particle size. The treatments, as well as the interaction between treatment and particle size, affected the pH of the litter (*p* = 0.0002 and *p* = 0.0004; respectively). In contrast, the pH of the litter was not affected by particle size (*p* > 0.05). Natural zeolite treatments, physical size, or their interaction did not affect litter moisture content (*p* > 0.05).

## 4. Discussion

Numerous studies have been conducted to reduce the use of antibiotics in the livestock and poultry industry, focusing on antibiotic alternatives to optimize growth performance and feed efficiency in livestock and poultry production [40]. Since 2007, zeolite-based additives have been declared safe for end users of meat, milk, or eggs from animals that received zeolite in feed or manure [41]. It was noted that clinoptilolite zeolite is registered by the European Community as a food additive under DIN 53 770.

Zeolites are traditionally used in the feed of broiler chickens as a technological additive to improve BWG and FCR [42]. Shariatmadari [10], and Schneider and Zimmermann [4] conducted a literature review on the use of zeolite in poultry production. Different zeolite contents in feed led to different results, which may affect the content of different nutrients and feed consistency. In addition, they addressed the issue of body weight gain. Our study found that the addition of natural zeolites (clinoptilolite) to broiler diets from day 1 to day 35 resulted in no significant differences in growth performance variables. This could be evidence of excessive zeolite content in broiler diets. These results are in agreement with those of [43], who concluded that natural and modified zeolites had no significant effect on broiler performance during the experiment. Schneider and Almeida [23] also found that 0.5% of zeolite supplementation had no effect on body weight (BW), BWG, feed intake (FI), or FCR throughout the experiment. In addition, the feed conversion of broilers receiving zeolite at any level was not affected [7]. The lack of BWG response to clinoptilolite supplementation is also consistent with the previous report by Zhou [44]. In contrast, Bilal, Kaygisiz [45] found that the addition of natural clinoptilolite to the diet had no effect on the BW and FCR of laying hens. However, some studies found that supplementing the diet with natural clinoptilolite improves the intestinal health status and productive performance and/or feed efficiency of the animals such as broilers [2,3,20,46,47,48,49]. Similarly, their beneficial effects on the turkey [22], Japanese quails [50], pigs [15,51], sheep [52,53], calves [52,54], and Nile tilapia (*Oreochromis niloticus*) [55]. Numerous studies in this field have found that using natural clinoptilolite improves the performance parameters of various animal species, which has been attributed to the type of the product used, its purity and physicochemical adsorption characteristics, and supplementation levels [56,57]. In contrast, some reports claimed that zeolite had a negative impact on BWG and FCR [58,59].

The particle size and the form of ground feed are crucial in determining FI, development of the digestive system, digestion, absorption of nutrients, intestinal health, and growth performance of poultry [60]. Using clinoptilolite with a physical size of 1 or 2 mm had no positive performance effects in this study. According to Parizadian, Kavan, Shams, and Shargh [42], using clinoptilolite with a physical size of 0.4–0.8 mm produces more beneficial performance effects than using other particle sizes. Natural zeolite particle sizes in broiler diets ranged from 0.1 mm to 1 mm [23,26]. In addition, Uriyanghai [27] found that using zeolite as grit in modern caged broilers with sizes ranging from 1–2.5 mm has no negative effect on broiler growth performance or gizzard development, but does result in slightly higher weight gain. Furthermore, Mery, Guerrero [28] investigated the effect of zeolite particle sizes of 0.5, 1.0, and 2.0 mm on microorganism adherence to zeolite. They found that a zeolite particle size of 0.5 mm had the highest ammonium adsorption capacity, which was 64% and 31% higher than particle sizes of 1.0 and 2.0 mm, respectively. There is evidence that coarser grinding results in a more uniform particle size, which improves the performance of birds that are fed mash diets [61,62,63]. This effect could be attributed to feed particle size’s beneficial effect on stimulating gizzard development and function as digestive contents. Increased grinding activity is associated with more developed gizzards, which leads to increased gut motility and nutrient digestion [61,63]. A large, well-developed gizzard improves gut motility by stimulating digestive enzyme production via increasing cholecystokinin release, which stimulates pancreatic enzyme secretion and gastro-duodenal refluxes [61,63]. Larger particles of feed may have been retained in the gastrointestinal (GI) tract for a longer period, potentially enhancing nutrient digestibility and energy utilization [60]. Broiler chickens fed coarse feed particles (897 μm) outperformed those fed fine particles (525 μm) in terms of growth performance [64].

Furthermore, the particle size, degree of accumulation, crystallite size, and particle porosity of zeolitic materials determine the access of ingesta fluids to the zeolitic surface during passage through the gastrointestinal tract and have a strong influence on its ion exchange, absorption, and catalytic activity [51]. Zeolites’ predictable effects may differ on factors such as nature, concentration, zeolite aluminum content, and calcium and phosphorus levels in the diet. There was no synergistic or antagonistic relationship between zeolite levels and broiler feed macromineral content. Zeolites inhibit P utilization by forming an indigestible compound with P via its aluminosilicate component, increase Ca usage, and indirectly impact P absorption and metabolism [11]. Zeolites may cause changes in element absorption, such as Ca and P, as well as electrolyte balance [65]. According to Azar, Adl [66], zeolite levels and physical sizes had no effect on serum Ca, P, Na, and Cl levels. Thus, these results are consistent with Nazifi and Dadras [67] who showed that supplementing broiler diets with natural zeolites (1.2%) had no effect on serum Mg, K, Na, Cl, and levels, but precise changes. On the other hand, Utlu and Celebi [68] found that dietary zeolite supplementation had no effect on plasma Ca, but did reduce the serum P concentration.

The effects of zeolites are being studied not only for changes in fowl performance but also for carcass traits and quality. There was no effect of zeolites, particle size, or their interactions on slaughter BW, carcass weight, or other selected carcass traits. As a result, no effect of zeolite addition on broiler carcass characteristics was demonstrated. The findings were consistent with our research. However, the zeolite–physical size interaction had a significant impact on the dressing percentage, with the dressing percentage decreasing as zeolite levels increased and physical particles became larger. Christaki–Sarikaki, Fortomaris [69] discovered a lower percentage of abdominal fat and higher thigh meat weight in the carcass, implying that 2.0% of natural zeolites in the broiler diet have a beneficial effect on fatty tissue deposition in birds.

There were no substantial differences in abdominal fat content in our study, but the average values were lower in the treatment group eating a 10 g zeolite/kg diet. The carcass characteristics agreed with Tatar, Boldaji’s [70] findings, who found that incorporating 2 or 4% natural zeolite into broiler diets resulted in no differences (*p* > 0.05) between treatments during the experiment. In addition, the inclusion of 0.5% zeolite in the diet did not lead to differences in the diet and had no effect on total carcass yield or broiler breast and leg meat [4]. Furthermore, the incorporation of 2% and 4% clinoptilolite in broiler basal diet had no effect on carcass weight, thigh, drumsticks, breast, abdominal fat, and distal back [71]. Moreover, a study by Prvulovic and Kojic [72] reported that spleen weight was higher in the 5% zeolite, whereas the weight of the other measured organs was not affected. Currently, the addition of zeolite to broiler chicken rearing could be proposed based on the similar carcass yield between zeolite-treated and zeolite-free treatments. However, given the scarcity of this type of scientific research, it is critical that it be continued.

The treatments, particle size, and their interaction had no effect on the percentage weights of lymphoid organs (*p* > 0.05). Similar to our study, there were no differences in internal organs such as the liver, spleen, heart, and bursa when adding natural zeolite [3,73]. There were no variations in internal organs such as the liver, heart, and bursa when zeolite was added to our study. Katouli, Boldaji [3] reported findings that were similar to ours. In contrast to Katouli, Boldaji [3], natural zeolite treatments had an effect on spleen proportional weight.

In our investigation, the total weight of the SI in treatments containing 1% zeolite was the highest (63.91 g) when compared to the control (53.79 g). The total length of the SI was the longest in treatments containing 0.5 percent zeolite (174.1 cm) and the shortest in treatments containing 1.5 percent zeolite (162.2 cm). Furthermore, zeolite particle size seemed to have no effect on the total or parts of the SI. This may be ascribed to clinoptilolite supplementation in diets increasing intestinal length and weight, improving intestinal morphology, and digestive enzyme activities of the gut such as amylase and protease [47]. Zeolite may influence the morphology and microbiota of the gut, the levels of apparent metabolizable energy (ME), and the digestibility of proteins [74]. The impacts of clinoptilolite on nutrient absorption were tested in broilers for beneficial changes in intestinal morphology, such as higher villi heights, and increases in digestive enzyme activity in the SI when diets are supplemented with natural zeolite (2.0%) [43].

Several factors, including genotype, rearing conditions, and feed additives, can influence muscle metabolism and chemical composition in poultry meat [75]. Zeolite inclusion in the rabbit feeding diet may improve the nutritional value of the meat [76]. Here, the interaction of clinoptilolite with particle size resulted in a change in the final pH of birds. Variations in meat pH can be attributed to changes in food additives, slaughter ecosystems, and animal management [22]. The use of natural zeolite as a turkey feed additive improves the antioxidative capacity of breast meat while also increasing polyunsaturated fatty acid levels in the body, resulting in better meat quality [22]. Furthermore, meat quality was assessed with a considerable increase in meat pH, indicating that the clinoptilolite could maintain meat quality for a longer period. The pH is known to affect the meat quality, with higher pH resulting in dark, firm, dry (DFD) meat, whereas lower pH results in pale, soft, exudative (PSE) meat due to denaturation of the proteins. Based on the meat quality values obtained, no chicken meat was classified as pale, soft, and exudative (PSE) meat, but it attempted to be dark, firm, and dry (DFD) meat. The threshold ranges to be considered are ≤ 5.8 (PSE), 5.9–6.2 (standard meat properties), and ≥6.3 (DFD) [77]. The results showed that the initial pH range is between (6.34 and 6.45) and the final pH range is between (5.81 and 5.83).

Furthermore, the inclusion of different particle sizes of zeolites had no influence on the color of the breast samples; however, higher decreased values in an initial and ultimate redness were observed with increasing zeolite dose-independent particle sizes. Smaller zeolite particles increased final lightness, whereas larger zeolite particles increased final yellowness. This is consistent with the findings of Biesek and Banaszak [59], who discovered that adding zeolite to the diet resulted in increased yellowness of the leg muscles of ducks. Individual study dissimilarities in the physicochemical traits of chicken muscles may be attributable to protein oxidation in the muscles, but material treatment after slaughter also has an impact [78].

WHC was unaffected by the treatments, particle size, or their interaction. This contradicts the findings of Biesek and Banaszak [59], who discovered that adding zeolite to breast muscles increased their water-holding capacity in six-week-old ducks. The WHC of breast samples depends on the content of oxidative muscle fibers [79]. Proteolysis in broiler breast muscles (pectoralis major) also has an effect on WHC [80]. In addition, natural zeolite treatments, particle size, and their interaction had no effect (*p* > 0.05) on the MFI and SF percent. The CL percent was affected by the natural zeolite treatments (*p* < 0.0001) and the treatment–article size interaction (*p* < 0.0001). For CL percent, breast muscle from birds fed 15 g/kg zeolite had the highest value, while breast muscle from birds fed 5 g/kg zeolite had the lowest value.

Here, breast muscle hardness varied from 5.76 to 7.25 N when the zeolite concentration increased from 0% to 2%, whereas Mallek and Fendri [81] found it varied from 1.53 to 2.76 N when the zeolite concentration increased from 0% to 1%. Chewiness, similar to hardness and elasticity, increases the addition of zeolite [81]. When zeolite was added, the compactness of the protein gel network was reduced, allowing for more water binding and tenderizing of the meat [81]. Here, chewiness was lowest in the birds fed 5 g zeolite/kg diets versus the control. Broiler chicken breast meat had less hardness, more springiness, and more cohesiveness in large particle size groups (2 mm).

According to Mallek, Fendri [81], zeolite supplementation in broiler ration improves performance, organoleptic variables, and, most importantly, Omega 3 fatty acid levels. Furthermore, numerous types of research have shown that silver nanoparticles (Ag-NPs) coated on zeolite are useful as feed additives due to their positive effects on FCR, meat quality, and hepatic enzymes in broilers [82]. Although it has the ability to enhance gut microflora, Ag-NPs may have side effects on the immune system of broilers [83].

The use of zeolites as soil ameliorants help to an improvement of soil physiochemical activities and in the reduction heavy metal toxicity. Due to their unique properties, zeolites improve fertilizer and water use efficiency and then reduce pollution by reducing nitrate leaching and nitrous oxide and ammonia emissions. The aforementioned characteristics significantly improve the growth, productivity, and quality of versatile crops while also maximizing resource use efficiency [84].

In our study, the pH of litter in treatments with 0.5% zeolite had the highest value (6.60), while treatments with 1% zeolite had the lowest value (5.76). Furthermore, zeolite supplementation had no effect on fecal moisture. In contrast to our findings, Nikolakakis, DOTAS [2] found that adding 2 to 3 percent natural zeolite to Cobb-500 broiler chicken feed improved litter quality. Fecal moisture was reduced in groups containing 3% zeolite [3]. Schneider, Zimmermann [4], on the other hand, found that feeding poultry a diet containing 0.5 percent zeolite reduced the pH and moisture of their excreta. Here, the pH of GIT decreased as particle size increased. A few authors such as Katouli, Boldaji [3] speculated that the structure of the mineral, the geographical origin of the zeolite, or its crystal size, structure, cavity shape, porosity, metal oxide content, environmental conditions, and animal species, could be to blame for these inconsistencies. One of the most critical challenges in chicken production was the control of litter nature. In the present poultry industry, the latter was seen as an advantage in terms of avoiding environmental and bird welfare issues as well as reducing productivity losses [85]. In addition, results indicate that the use of clinoptilolite as a feed additive contributes to a rise in ammonia emissions. This could be due to the ammonia-binding impact of natural zeolites in the digestive tract of broilers or to an increase in total protein intake [46]. Another study by Eleroğlu and Yalçın [86] showed lower average levels of moisture in clinoptilolite-supplemented litter (25, 50, and 75% of the total litter or bedding volume) in broilers. Dietary zeolite supplementation at 2% optimizes performance and particularly P digestibility, which may lead to a reduction in lamb waste and, as a result, less pollution of the environment [53]. As a result, it is concluded that natural clinoptilolite could be used in alternative management strategies; however, other considerations, such as the type of natural clinoptilolite or its incorporation ratio, need to be investigated further. This study proposed that zeolites be added to the litter rather than the diet to reduce excess litter moisture, which is detrimental to broiler performance. Furthermore, the results of this study suggested that zeolite at concentrations of up to 10 g zeolite/kg diet be added to broiler diets to improve broiler performance.

## 5. Conclusions

The present study concluded that dietary zeolite is not effective in either increasing broiler productive performance or maintaining litter quality at current levels. However, the zeolite may be improved numerically (*p* > 0.05) in performance and production efficiency factor with increasing doses up to 10 g zeolite/kg diet. However, from a 10–20 g zeolite/kg diet, the performance and production efficiency were adversely affected. According to current data, using zeolite in broiler diets at doses greater than 10 g zeolite/kg diet is not recommended due to adverse effects on performance and production efficiency when compared to lower doses. More research is needed to determine the safety concerns of zeolite broiler chickens at higher doses.

## Figures and Tables

**Table 1:** Diet composition and calculated nutrient content (%).

Ingredients	Experimental Diet	
	Starter (0–14 Days)	Finisher (15–35 Days)
Yellow corn	57.25	58.41
Soybean meal	30.00	32.91
Corn gluten	6.00	0.00
Palm oil	2.19	4.56
Dicalcium phosphate	2.30	1.57
Limestone	0.70	1.01
Salt	0.45	0.55
Min Vit Premix 0.5% ^a^	0.50	0.50
DL-Methionine	0.18	0.29
Lysine-HCL	0.32	0.12
Threonine	0.11	0.03
Choline C170	0.05	0.05
Total	100	100
Calculated analysis		
Metabolic energy (ME) (Kcal/kg)	3000	3200
Crude protein (%)	23	20
D-Lysine (%)	1.28	1.21
DL-Methionine (%)		0.73
TSAA (%)	0.95	0.86
Calcium (%)	0.96	0.87
D-Threonine (%)	0.86	0.70
Non-phytate P (%)	0.47	0.39

^a^ Containing by kg of diets: vitamin A—2,400,000 IU; vitamin D—1,000,000 IU; vitamin E—16,000 IU; vitamin K—800 mg; vitamin B1—600 mg; vitamin B2—1600 mg; vitamin B6—1000 mg; vitamin B12—6 mg; niacin—8000 mg; folic acid—400 mg; pantothenic acid—3000 mg; biotin—40 mg; antioxidant—3000 mg; cobalt—80 mg; copper—2000 mg; iodine—400; iron—1200 mg; manganese—18,000 mg; selenium—60 mg; and zinc—14,000 mg.

**Table 2:** Effect of zeolite supplementation and particle size on body weight (g) and body weight gain (g) of broiler chickens.

Main Effects	Body Weight (g)			Body Weight Gain (g)		
	1 d	21 d	35 d	1–21 d	22–35 d	1–35 d
Treatments ^1^ (TR):						
Control	42.20	708.42	1838.69	666.20	1130.27	1796.47
T1	42.18	738.82	1884.24	696.64	1145.43	1842.06
T2	42.18	749.15	1890.12	706.97	1140.98	1847.95
T3	42.19	725.22	1842.36	683.03	1117.15	1800.17
T4	42.18	712.57	1852.82	670.38	1140.26	1810.64
SEM ^2^	0.017	17.46	22.57	17.47	17.92	22.57
Particle Size (PS):						
1 mm	42.18	729.77	1856.73	687.58	1126.97	1814.55
2 mm	42.18	723.90	1866.56	681.71	1142.66	1824.37
SEM ^2^	0.010	11.04	14.27	11.05	11.33	14.27
Source of variation	*p*-value					
TR	0.440	0.429	0.351	0.428	0.808	0.351
PS	0.896	0.708	0.628	0.708	0.331	0.628
TR × PS	0.571	0.862	0.198	0.862	0.075	0.198

^1^ Treatments: Control, T1, T2, T3, and T4 = 0, 5, 10, 15, and 20 g of zeolite per kg of diet, respectively. *n* = 16 replicate/treatment, *n* = 8 replicate/particle size of each treatment. ^2^ SEM = standard error of means for each main effect.

**Table 3:** Effect of zeolite supplementation and particle size on total feed intake (g), feed conversion ratio (g:g), and the production efficiency factor of broiler chickens.

Main Effects	TFI (g)			FCR (g:g)			PEF	
	1–21 d	22–35 d	1–35 d	1–21 d	22–35 d	1–35 d	21 d	35 d
Treatments ^1^ (TR):								
Control	832.83	1657.88	2490.71	1.25	1.46	1.38	406.91	379.20
T1	888.44	1697.26	2585.69	1.27	1.48	1.40	414.21	383.91
T2	897.72	1663.99	2561.71	1.27	1.45	1.38	421.80	390.08
T3	872.44	1671.60	2544.04	1.28	1.50	1.41	407.34	372.71
T4	874.63	1653.93	2528.56	1.30	1.45	1.39	390.72	379.58
SEM ^2^	16.71	17.53	28.06	0.016	0.020	0.011	14.77	6.86
Particle Size (PS):								
1 mm	873.99	1661.28	2535.27	1.27	1.47	1.39	410.66	380.02
2 mm	872.43	1676.58	2549.02	1.28	1.47	1.39	405.73	382.17
SEM ^2^	10.57	11.09	17.74	0.010	0.012	0.007	9.34	4.34
Source of variation	*p*-value							
TR	0.078	0.435	0.179	0.342	0.516	0.365	0.659	0.484
PS	0.917	0.333	0.585	0.409	0.967	0.960	0.709	0.727
TR × PS	0.198	0.611	0.415	0.636	0.076	0.186	0.962	0.148

^1^ Treatments: Control, T1, T2, T3, and T4 = 0, 5, 10, 15, and 20 g of zeolite per kg of diet, respectively. TFI = total feed intake; FCR = feed conversion ratio and PEF = the production efficiency factor. *n* = 16 replicate/treatment, *n* = 8 replicate/particle size of each treatment. ^2^ SEM = standard error of means for each main effect.

**Table 4:** Effect of zeolite supplementation and particle size on the carcass traits of 35-day-old broiler chickens.

Main Effects	LW (g)	CW (g)	Dressing (%)	Breast (%)	Legs (%)	Gizzard (%)	Liver (%)	Heart (%)	Pancreas (%)	AF (%)
Treatments (TR):										
Control	1749.5	1129.0	64.76	41.73	40.07	3.93	3.18	0.73	0.38	1.78
T1	1819.5	1174.2	64.49	40.32	39.84	3.95	3.32	0.73	0.40	1.47
T2	1883.2	1212.8	64.38	42.35	39.74	3.99	3.23	0.72	0.40	1.39
T3	1782.9	1142.8	64.08	41.01	40.16	3.84	3.17	0.71	0.39	1.79
T4	1773.0	1126.2	63.36	40.82	40.58	4.03	3.32	0.70	0.39	1.70
SEM ^1^	35.04	26.72	0.48	0.56	0.42	0.15	0.13	0.02	0.01	0.12
Particle Size (PS):										
1 mm	1803.3	1162.5	64.48	41.11	40.19	3.96	3.25	0.73	0.39	1.59
2 mm	1799.9	1151.5	63.94	41.38	39.97	3.93	3.24	0.70	0.39	1.65
SEM ^1^	22.16	16.90	0.30	0.36	0.26	0.10	0.08	0.01	0.01	0.07
Source of variation	*p*-value									
TR	0.078	0.126	0.319	0.111	0.667	0.940	0.881	0.860	0.943	0.078
PS	0.914	0.647	0.228	0.610	0.557	0.829	0.971	0.186	0.919	0.588
TR × PS	0.240	0.142	0.019	0.196	0.851	0.321	0.141	0.150	0.012	0.270

Control, T1, T2, T3, and T4 = 0, 5, 10, 15, and 20 g of zeolite per kg of diet, respectively. ^1^ SEM = standard error of means for each main effect. LW = live weight; CW = carcass weight; and AF = abdominal fat.

**Table 5:** Effect of zeolite supplementation and particle size on relative weights of lymphoid organs in broiler chickens aged 35 days.

Main Effects	Thymus (%)	Bursa (%)	Spleen (%)
Treatments (TR):			
Control	1.526	0.475	0.475 ^a^
T1	1.581	0.607	0.428 ^ab^
T2	1.411	0.464	0.361 ^b^
T3	1.218	0.495	0.345 ^b^
T4	1.545	0.470	0.346 ^b^
SEM ^1^	0.174	0.046	0.030
Particle Size (PS):			
1 mm	1.479	0.516	0.413
2 mm	1.433	0.488	0.369
SEM ^1^	0.110	0.029	0.019
Source of variation	*p*-value		
TR	0.585	0.166	0.011
PS	0.769	0.497	0.118
TR × PS	0.841	0.265	0.197

Control, T1, T2, T3, and T4 = 0, 5, 10, 15, and 20 g of zeolite per kg of diet, respectively. Means values within columns with ^a^ and ^b^ superscripts are significantly different (*p* < 0.05). ^1^ SEM = standard error of means for each main effect.

**Table 6:** Effect of zeolite supplementation and particle size on pH and temperature of meat components of 35-day-old broiler chickens.

Main Effects	pH		Temperature (°C)	
	Initial	Ultimate	Initial	Ultimate
Treatments (TR):				
Control	6.44	5.83	27.46 ^b^	21.86 ^c^
T1	6.39	5.82	27.15 ^c^	22.56 ^a^
T2	6.45	5.82	27.38 ^bc^	22.77 ^a^
T3	6.34	5.81	28.41 ^a^	22.65 ^a^
T4	6.41	5.82	28.29 ^a^	22.38 ^a^
SEM ^1^	0.030	0.015	0.090	0.129
Particle Size (PS):				
1 mm	6.42	5.81	27.45 ^b^	22.47
2 mm	6.39	5.83	28.02 ^a^	22.42
SEM ^1^	0.019	0.009	0.057	0.082
Source of variation	*p*-value			
TR	0.099	0.854	<0.0001	<0.0001
PS	0.252	0.120	<0.0001	0.622
TR × PS	0.070	<0.0001	<0.0001	0.164

Control, T1, T2, T3, and T4 = 0, 5, 10, 15, and 20 g of zeolite per kg of diet, respectively. Means values within columns with different superscripts (a–c) are significantly different (*p* < 0.05). ^1^ SEM = standard error of means for each main effect. Initial, measured after slaughter. Ultimate, measured after 24 h.

**Table 7:** Effect of zeolite supplementation and particle size on the color of meat components of 35-day-old broiler chickens.

Main Effects	Initial Color Components			Ultimate Color Components		
	L*	a*	b*	L*	a*	b*
Treatments (TR):						
Control	41.54	6.32 ^ab^	7.47	43.04	7.08 ^b^	11.53
T1	41.05	7.28 ^a^	7.18	41.63	8.41 ^a^	10.98
T2	41.70	6.32 ^ab^	6.97	43.13	7.56 ^ab^	11.84
T3	41.80	5.65 ^bc^	7.24	43.01	6.92 ^b^	11.53
T4	42.60	5.06 ^c^	6.84	43.62	5.73 ^c^	11.71
SEM ^1^	0.577	0.368	0.318	0.572	0.392	0.386
Particle Size (PS):						
1 mm	42.02	6.25	7.03	44.04 ^a^	7.01	11.10 ^b^
2 mm	41.46	6.00	7.25	41.74 ^b^	7.26	11.94 ^a^
SEM ^1^	0.365	0.233	0.201	0.362	0.247	0.244
Source of variation	*p*-value					
TR	0.443	0.001	0.680	0.162	0.0003	0.581
PS	0.284	0.445	0.457	<0.0001	0.478	0.017
TR×PS	0.138	0.012	0.215	0.0004	<0.0001	0.752

Control, T1, T2, T3, and T4 = 0, 5, 10, 15, and 20 g of zeolite per kg of diet, respectively. Means values within columns with different superscripts (a–c) are significantly different (*p* < 0.05). ^1^ SEM = standard error of means for each main effect. Initial, measured after slaughter; ultimate, measured after 24 h; L, Lightness; a, Redness; b, Yellowness.

**Table 8:** Effect of zeolite supplementation and particle size on the meat quality of 35-day-old broiler chickens.

Main Effects	CL (%)	WHC (%)	MFI	SF (N)	Texture Profile Analysis (TPA)			
					HR	SI	CI	CHI
Treatments (TR):								
Control	22.07 ^cd^	28.53	100.24	7.25	10.14	0.65	0.44	2.77 ^a^
T1	20.28 ^d^	28.71	89.90	6.34	7.60	0.66	0.42	2.10 ^b^
T2	27.37 ^ab^	29.52	76.63	5.76	8.99	0.64	0.40	2.45 ^ab^
T3	29.43 ^a^	28.93	81.56	7.09	9.72	0.66	0.43	2.79 ^a^
T4	24.91 ^bc^	27.19	72.53	6.01	8.30	0.67	0.42	2.32 ^ab^
SEM ^1^	0.986	1.708	7.732	0.669	0.677	0.012	0.010	0.160
Particle Size (PS):								
1 mm	24.33	29.43	79.65	6.47	9.83 ^a^	0.63 ^b^	0.40 ^b^	2.50
2 mm	25.29	27.72	88.69	6.51	8.07 ^b^	0.68 ^a^	0.45 ^a^	2.47
SEM ^1^	0.623	1.080	4.890	0.423	0.428	0.008	0.006	0.101
Source of variation	*p*-value							
TR	<0.0001	0.905	0.115	0.446	0.083	0.835	0.405	0.022
PS	0.284	0.274	0.202	0.942	0.007	0.0004	<0.0001	0.835
TR × PS	<0.0001	0.212	0.268	0.918	0.184	0.715	0.082	0.080

Control, T1, T2, T3, and T4 = 0, 5, 10, 15, and 20 g of zeolite per kg of diet, respectively. Means values within columns with different superscripts (a–d) are significantly different (*p* < 0.05). ^1^ SEM = standard error of means for each main effect. CL = cooking loss; WHC = water holding capacity; MFI = myofibril fragmentation index; SF = Shear Force; HR = Hardness; SI = springiness index; CI = cohesiveness index and CHI = chewiness index.

**Table 9:** Effect of zeolite supplementation and particle size on small intestinal morphometric of 35-day-old broiler chickens.

Main Effects	Weight				Length			
	Total (g)	Duodenum (%)	Jejunum (%)	Ileum (%)	Total (cm)	Duodenum (%)	Jejunum (%)	Ileum (%)
Treatments (TR):								
Control	53.79 ^c^	18.71	42.27 ^b^	39.01	172.6 ^a^	16.50	42.22	41.26
T1	61.30 ^ab^	17.00	45.95 ^a^	37.03	174.1 ^a^	16.37	42.53	41.09
T2	63.91 ^a^	17.91	43.09 ^b^	38.99	172.9 ^a^	17.33	41.36	41.30
T3	57.62 ^bc^	18.02	43.80 ^b^	38.16	162.2 ^b^	16.60	42.08	41.31
T4	59.04 ^abc^	17.02	43.37 ^b^	39.59	168.2 ^ab^	16.19	42.30	41.50
SEM ^1^	2.035	0.553	0.736	0.982	2.393	0.316	0.419	0.499
Particle Size (PS):								
1 mm	60.45	18.20	43.81	37.97	170.2	16.77	42.11	41.11
2 mm	57.82	17.26	43.58	39.15	169.7	16.43	42.09	41.47
SEM ^1^	1.287	0.349	0.465	0.621	1.514	0.20	0.265	0.316
Source of variation	*p*-value							
TR	0.011	0.150	0.011	0.400	0.004	0.113	0.364	0.986
PS	0.153	0.061	0.722	0.186	0.824	0.223	0.952	0.429
TR × PS	<0.0001	<0.0001	0.0004	0.082	0.003	<0.0001	0.117	0.083

Control, T1, T2, T3, and T4 = 0, 5, 10, 15, and 20 g of zeolite per kg of diet, respectively. Means values within columns with different superscripts (a–c) are significantly different (*p* < 0.05). ^1^ SEM = standard error of means for each main effect.

**Table 10:** Effect of zeolite supplementation and particle size on pH of excreta/litter and moisture content of litter of 35-day-old broilers.

Main Effects	pH		Excreta/Litter Moisture %
	Gizzard	Litter	
Treatments (TR):			
Control	6.676	5.771 ^c^	15.866
T1	6.716	6.595 ^a^	16.755
T2	6.676	5.763 ^c^	17.027
T3	6.813	6.333 ^ab^	17.105
T4	6.712	6.047 ^bc^	15.974
SEM ^1^	0.058	0.142	0.975
Particle Size (PS):			
1 mm	6.772 ^a^	6.178	16.167
2 mm	6.665 ^b^	6.025	16.923
SEM ^1^	0.036	0.090	0.617
Source of variation	*p*-value		
TR	0.451	0.0002	0.835
PS	0.043	0.233	0.389
TR × PS	0.086	0.0004	0.056

Control, T1, T2, T3, and T4 = 0, 5, 10, 15, and 20 g of zeolite per kg of diet, respectively. Means values within columns with different superscripts (a–c) are significantly different (*p* < 0.05). ^1^ SEM = standard error of means for each main effect.

## Data Availability

Not applicable.
